# Early detection of soybean mosaic virus using portable Raman spectroscopy coupled with machine learning

**DOI:** 10.3389/fpls.2025.1750535

**Published:** 2026-01-06

**Authors:** Yujia Han, Hongpu Guan, Dagang Wang, Yafei Zhang, Weixuan Zhang, Yiming Zhao, Longgang Zhao, Zhaohua Wang, Tingting Wu, Yanru Zhao, Hexiang Luan

**Affiliations:** 1 Qingdao Agricultural University, Qingdao, Shandong, China; 2 College of Mechanical and Electronic Engineering, Northwest A & F University, Yangling, Shaanxi, China; 3 Crop Institute of Anhui Academy of Agricultural Sciences, Hefei, Anhui, China; 4 Institute of Agricultural Information and Economics, Shandong Academy of Agricultural Sciences, Jinan, China

**Keywords:** early detection, Raman spectroscopy, SMV-SC3, soybean, virus infection

## Abstract

**Introduction:**

Soybean mosaic virus (SMV) is one of the major pathogens affecting global soybean yield and quality, and its early and accurate detection is essential for disease warning and precision management. This study proposes a non-invasive early detection method by integrating portable Raman spectroscopy with artificial intelligence algorithms.

**Methods:**

Raman spectra of leaves from both resistant and susceptible soybean cultivars were collected at different infection stages (0, 2, 4, and 6 days post-inoculation), and preprocessed using Savitzky–Golay (S-G) smoothing and adaptive iteratively reweighted penalized least squares (Air-PLS) baseline correction. Four classification models—1D-CNN, SVM, KNN, and BP-ANN—were developed to classify samples from different infection stages.

**Results:**

Spectral feature analysis revealed significant changes in carotenoid levels caused by viral infection, and distinct spectral responses between resistant and susceptible cultivars during disease progression. Among the four classification models, the 1D-CNN model achieved the highest prediction accuracy of 90%. In addition, principal component analysis (PCA) indicated that the Raman spectroscopy-based method significantly advanced the early detection of SMV (SC3) to 4 days post-inoculation, compared to 7–10 days required by conventional methods.

**Discussion:**

This evidences the superior capability of Raman spectroscopy for monitoring the dynamics of SMV infection and its potential to considerably reduce the duration of diagnosis. This study confirms the feasibility and efficiency of Raman spectroscopy combined with deep learning for in situ early detection of plant viral diseases and provides a promising reference for non-destructive diagnosis of early-stage foliar infections.

## Introduction

Soybean, a globally significant triple-purpose crop for oil extraction, grain production, and animal feed, holds strategic importance in national socio-economic development. However, soybeans are highly susceptible to pathogens during vegetative growth. Among these, Soybean Mosaic Virus (SMV) is particularly devastating, causing substantial yield and quality losses (Hill, 2003; Hill and Whitham, 2014). Traditional methods for detecting SMV play a significant practical role in soybean disease control and prevention. Their value lies primarily in economic efficiency and practicality: low-cost techniques such as symptom observation and ELISA test strips enable farmers to rapidly screen for infections and implement control measures like removing infected plants and crop rotation. These methods also provide phenotypic screening criteria for disease-resistant breeding while fostering the accumulation of empirical knowledge linking disease manifestations to specific symptoms. However, traditional approaches exhibit notable limitations. For example, ELISA lacks sufficient sensitivity for samples with low viral concentrations, often missing early-stage infections; symptom-based diagnoses are prone to misjudgment due to interference from environmental stressors or nutrient deficiencies; and biological inoculation methods require weeks to yield results. Furthermore, delayed detection limits timely warnings, often causing missed opportunities during critical control periods. Although traditional techniques remain vital for disease management and foundational research, their shortcomings in accuracy and efficiency highlight the urgent need for AI-driven technologies to establish a more robust and responsive disease control system.

In recent years, advances in artificial intelligence technology have enabled the integration of spectroscopy and intelligent algorithms, offering a novel pathway for plant disease detection. This combined approach not only overcomes the limitations of traditional methods, such as being time-consuming and reliant on empirical judgment, but also significantly enhances detection efficiency and reduces costs (Feng et al., 2020). At present, the development of portable spectroscopic devices has facilitated the field and practical process of plant disease monitoring technology in complex environments. It has the advantages of small size, simple operation and fast response speed, which can carry out *in situ* data collection and instant analysis without damage to plant tissues, providing a technical basis for the realization of early warning and precise prevention and control of crop diseases (Saletnik et al., 2024; Orecchio et al., 2025).

Raman spectroscopy is characterized by its insensitivity to water, non-destructiveness, and high precision, enabling the direct retrieval of biochemical information from plant tissues without complex pretreatment. It can accurately identify plant metabolites and cell wall structural changes caused by pathogen infection in the early stage of diseases while ensuring the integrity of samples (Payne and Kurouski, 2021), which has certain potential and advantages in plant disease detection. Raman spectroscopy realizes the accurate analysis of material composition through the fingerprint peaks generated by molecular vibration. The peak position shift reflects the vibration mode of molecular bonds, and the peak intensity change indicates the variation in polarizability. Further combined with artificial intelligence algorithms, Raman spectroscopy enables the qualitative and quantitative detection of key substances during plant disease infection.

Portable Raman devices integrated with artificial intelligence (AI) algorithms have been successfully employed to diagnose various plant diseases. Sanchez et al. applied portable Raman spectroscopy to the detection of tomato Ribery disease, and combined Raman data with partial least squares discriminant analysis (PLS-DA) to realize the quantitative diagnosis of LsoA and LsoB in tomato plants (Sanchez et al., 2020). Zhao et al. collected Raman spectral curves of healthy and infected Sclerotinia rape leaves. The Least squares Support Vector Machine (LS-SVM) machine learning model was established, and the recognition rate reached 100%. When Perez et al. used Raman spectroscopy to detect citrus greening disease, PCA was used for clustering analysis, and LDA was used to classify and model the analyzed data, with an accuracy of 89.2% (Perez et al., 2016). Xue et al. proposed a deep learning model combining Long Short-Term memory network (LSTM) and convolutional neural network (CNN) to realize the non-destructive quantitative detection of chlortick residues in corn oil by Raman spectroscopy. Their proposed CNN-LSTM fusion model achieved a predictive accuracy with an RMSEP of 12.3 mg·kg^-^¹ and an R² value of 0.90 (Egging et al., 2018). Existing studies have shown that portable Raman devices combined with artificial intelligence algorithms can perform non-destructive, fast and accurate identification of early plant diseases. It provides key technical support and theoretical basis for promoting the application of precise monitoring of plant diseases in the direction of agricultural intelligence.

**Figure 1:**
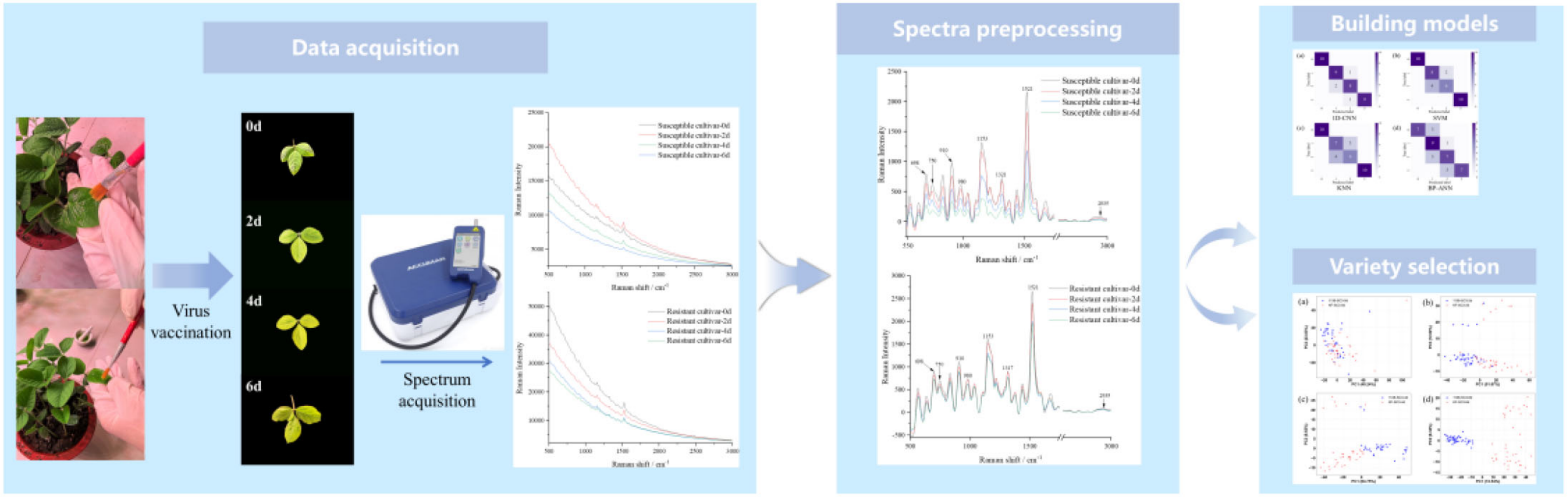
Main steps for early detection of SMV using portable Raman spectroscopy combined with artificial intelligence algorithms.

In this study, Raman spectroscopy was combined with artificial intelligence algorithms to develop an early detection model for SMV, and resistant soybean varieties were further screened. The experimental workflow comprised three main stages: Firstly, following preparation of SMV-infected soybean plants with leaves in optimal condition, Raman spectral data were collected. Secondly, Savitzky-Golay (S-G) smoothing filtering method and adaptive iteratively reweighted penalized least squares (Air-PLS) were used to preprocess the Raman spectral data. Finally, four multi-class classification models including 1D-CNN, SVM, KNN and BPNN were established and compared to realize the early detection of SMV. Meanwhile, to better identify disease-resistant soybean varieties, principal component analysis (PCA) was employed to observe the spectral variations between resistant and susceptible cultivars at different infection stages, enabling more effective discrimination between the two types. This study provides valuable guidance for both the early detection of SMV and the screening of resistant soybean cultivars. The main research process is depicted in **Figure 1**.

## Materials and methods

### Experimental material

The SMV strain was maintained on disease-susceptible variety NN1138-2. SMV inoculation was performed on the true leaves at the emergence stage of the first trifoliate leaf pair under controlled conditions in an aphid-free greenhouse with day and night temperatures of 25 °C and 20 °C, respectively. The inoculation was performed mechanically as described earlier (Luan et al., 2016). Briefly, leaves of the susceptible soybean cultivar NN1138–2 and the resistant cultivar KF were inoculated with an inoculum prepared by triturating the SMV SC3 strain. The trituration was performed using a sterile pestle in a pre-chilled mortar with 0.01 M phosphate buffer (pH 7.2; buffer-to-tissue ratio 4:1–5:1 v/w).

### Test instrument - parameters

Raman spectra were acquired using a portable Raman spectrometer (Accuman PR-500, Ocean Optics). After repeated experimental trials, the laser used in this study was a solid-state laser with an output wavelength of 785 nm. The Raman spectrum measurement range was 200~3900cm^-1^, the laser power was set as 5mW, and the integration time was 0.2s.

### Raman spectroscopy data processing methods

After collecting the raw Raman spectra, the data are susceptible to the interference of background noise and the ‘baseline drift’ phenomenon. Common methods for Raman spectrum signal interference removal include: baseline correction using techniques such as polynomial fitting and wavelet transform; noise reduction through methods like wavelet denoising and S-G filtering; cosmic ray removal via median filtering; and feature peak extraction optimized by PCA or machine learning algorithms. In this experiment, the S-G smoothing filter noise reduction method is mainly used to preprocess the data. The S-G filtering denoising method mainly uses the local polynomial fitting to preserve the spectral characteristic peak while using the sliding window convolution to eliminate the high-frequency noise, which is suitable for the signal optimization of Raman spectroscopy, so as to retain more effective spectral information.

Adaptive Iteratively Reweighted Penalized Least Squares (Air-PLS) was used for baseline correction, which dynamically fitted the baseline of Raman spectra by iteratively optimizing the weighting function and penalty term. Its key advantage lies in its ability to autonomously adapt smoothing parameters and noise weights, thereby effectively suppressing intense fluorescence background while preventing distortion of characteristic peak signals. It is suitable for automatic correction of high noise and complex baseline (Xu et al., 2021).

### Sample size design and distribution

This study systematically collected spectral data at multiple predetermined time points post-virus inoculation to capture the early dynamics of Soybean Mosaic Virus (SMV) infection. For the susceptible cultivar NN1138–2 and the resistant cultivar KF, 100 plants were selected as biological replicates at each infection time point (0 d, 2 d, 4 d, and 6 d) to account for individual variation. The first pair of trifoliate leaves was selected from each plant, and three spectral measurements were taken from the same leaf region as technical replicates to minimize measurement error. In total, the study obtained 2,400 valid samples across all experimental groups, with 1,200 samples each from NN1138–2 and KF, evenly distributed across the healthy stage (0 d) and the three infection stages (2 d, 4 d, 6 d).

### Building machine learning models - criteria for model discrimination

Convolutional neural network (CNN) is a conventional deep learning algorithm. Its core structure contains a convolutional layer, a pooling layer and a fully connected layer. The convolutional layer extracts spatial features through local perception and parameter sharing, the pooling layer reduces dimension and improves robustness, and the fully connected layer realizes classification. In spectral analysis and applications, a one-dimensional convolution kernel is mainly used to extract local features of spectral signals (such as near-infrared spectra and Raman spectra) and to automatically identify key information including peak positions and waveforms. This method is suitable for material composition identification, pollutant detection, soil quality analysis, and other agricultural-related fields (Chen et al., 2024). The advantage of SVM is that it can deal with high-dimensional and nonlinear data. In spectral analysis, SVM uses kernel functions (such as RBF) to map data to high-dimensional space, and solves the classification problem of complex features of near-infrared and Raman spectra. Its advantage lies in its strong generalization ability under small samples, and it can effectively distinguish spectral subtle differences (Sharma et al., 2023).

K-Nearest Neighbors (KNN) is an instance-based supervised learning algorithm that computes the distance between samples for classification or regression. In spectral analysis, KNN uses the similarity of spectral data to predict the attributes of unknown samples according to the category or value of neighboring samples, which is suitable for substance classification and qualitative analysis of components. The advantage of this algorithm is that it is training-free and simple and intuitive, but it is computationally intensive and sensitive to high-dimensional noise. This is often combined with dimensionality reduction (such as PCA) or feature selection optimization (Tian et al., 2020).

Back Propagation Artificial Neural Network (BP-ANN) is a multilayer feedforward network, which is trained by the error back propagation algorithm. Its structure includes input layer, hidden layer and output layer, and uses activation function to realize nonlinear mapping. The core mechanism is to adjust the weights and biases by gradient descent and backpropagate the output error layer by layer to minimize the loss function. On this basis, the classification of spectral data is realized.

### Model evaluation metrics

To assess the effectiveness of the developed models, classification accuracy, loss, and confusion matrix metrics—including precision, recall, F1-score, and overall accuracy—were utilized. These evaluation criteria are commonly applied in both machine learning and deep learning studies. The detailed mathematical definitions of each core metric are given in the subsequent formulas: precision is calculated as (Equation 1), recall is defined as (Equation 2), the F1-score is derived from (Equation 3), and the overall accuracy is computed according to (Equation 4).

(1) $$Accuracy=\frac{TP+TN}{TP+TN+FP+FN}$$

(2) $$Precision=\frac{TP}{TP+FP}$$

(3) $$Recall=\frac{TP}{TP+FN}$$

(4) $$F1-score=2\frac{Precisin\times Recall}{Precisin+Recall}$$

TP refers to the number of positive samples correctly identified by the model, while TN denotes the number of correctly predicted negative samples. FP represents the number of positive cases incorrectly classified as negative, and FN indicates negative samples misclassified as positive.

### Cluster analysis

Principal Component Analysis (PCA) is a widely used dimensionality reduction technique that transforms high-dimensional data into a small number of principal components through linear transformation, aiming to retain as much variance from the original dataset as possible and extract the most representative features. In this study, PCA was applied to classify resistant and susceptible soybean cultivars. The PCA procedure was conducted based on the covariance matrix, using the full-range Raman spectral data as input. The variance explained plot was employed to visualize the distribution of spectral features and assess their contributions across the Raman wavelengths.

## Results and discussion

### Raman spectral curves and their fingerprint analysis

**Figure 2:**
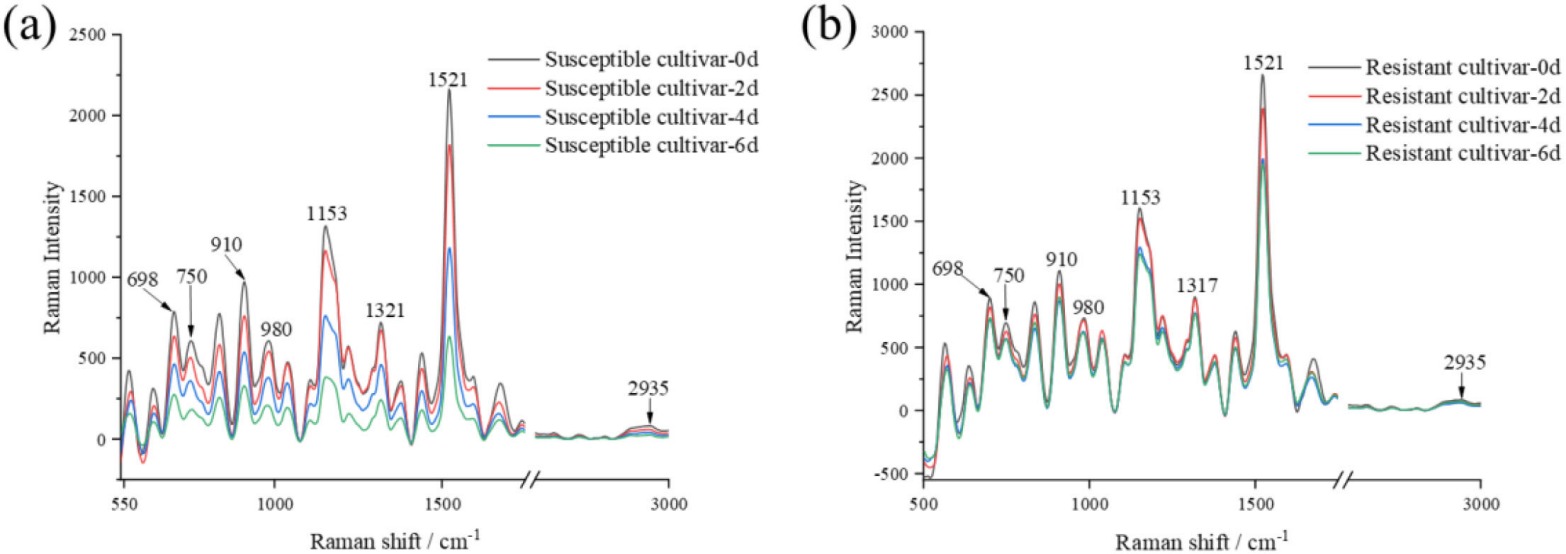
The average Raman spectra of 1138-SC3 (susceptible) and KF-SC3 (resistant) soybean leaves. **(a)** 1138-SC3 (Susceptible cultivar). **(b)** KF-SC3 (Resistant cultivar). Spectra are shown for healthy (0 d) and SMV-infected leaves at 2, 4, and 6 days post-inoculation.

The raw Raman spectra are first processed by S-G smoothing and denoising. The smoothed data were then processed by Air-PLS baseline correction. **Figure 2** shows the average Raman spectra of the treated 1138 quality SC3 virus inoculated leaves and the KF variety SC3 virus inoculated leaves at different infection times (0 d, 2 d, 4 d, 6 d):

The figure depicts the changes in the Raman spectra of soybean leaves from the 1138 and KF varieties at 0, 2, 4, and 6 days following SMV infection. It can be observed that there are many characteristic Raman bands in the 500-1,600 cm^-1^ range. Among them, Raman peaks at 698 cm^-1^ and 750 cm^-1^ are assigned to the skeletal bending vibration of lignin. The Raman peak at 910 cm^-1^ is correlated with H-C-C and H-C-O bending vibrations at the C6 position in cellulose molecules. The peak at 980 cm^-1^ is caused by the oscillating vibration of CH3 in the carotenoid plane and chlorophyll molecules in the leaf tissue. The peak at 1,153 cm^-^¹ is more prominent and is attributed to the C=C stretching vibration of carotenoids. The peak at 1,521 cm^-^¹ is the most prominent and intense, resulting from the C-C stretching vibration of carotenoids. **Table 1** specifically shows the distribution of Raman spectral characteristic peaks of soybean leaves:

**Table 1:** Assignment of characteristic Raman shifts.

Raman shift/cm^-1^	Vibrational assignment	References
698	Skeletal deformation vibrations of lignin	(Agarwal et al., 2011)
750		
910	The bending vibrations of HCC and HCO at C6 in cellulose	(Adapa et al., 2009)
980	The in-plane CH_3_ rocking vibration of carotenoids	(Gill et al., 1970)
1,153	The C-O-C vibration of carotenoids	(Gill et al., 1970)
1,321	The C-H stretching vibration of cellulose	(Agarwal et al., 2011)
1,521	The C=C stretching vibration of carotenoids	(Koyama et al., 1986)

**Figure 3:**
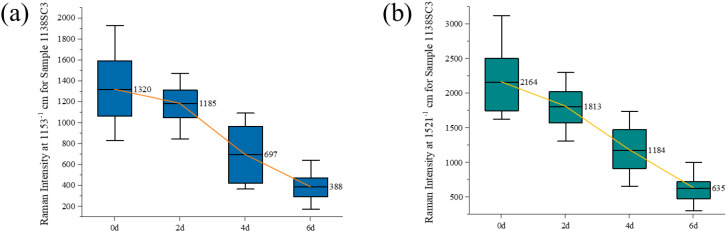
Intensity dynamics of key Raman peaks in susceptible soybean leaves during SMV infection. **(a)** Peak at 1,153 cm^-^¹. **(b)** Peak at 1,521 cm^-^¹. Data are from cultivar 1138 at 0, 2, 4, and 6 days post-inoculation.

**Figure 4:**
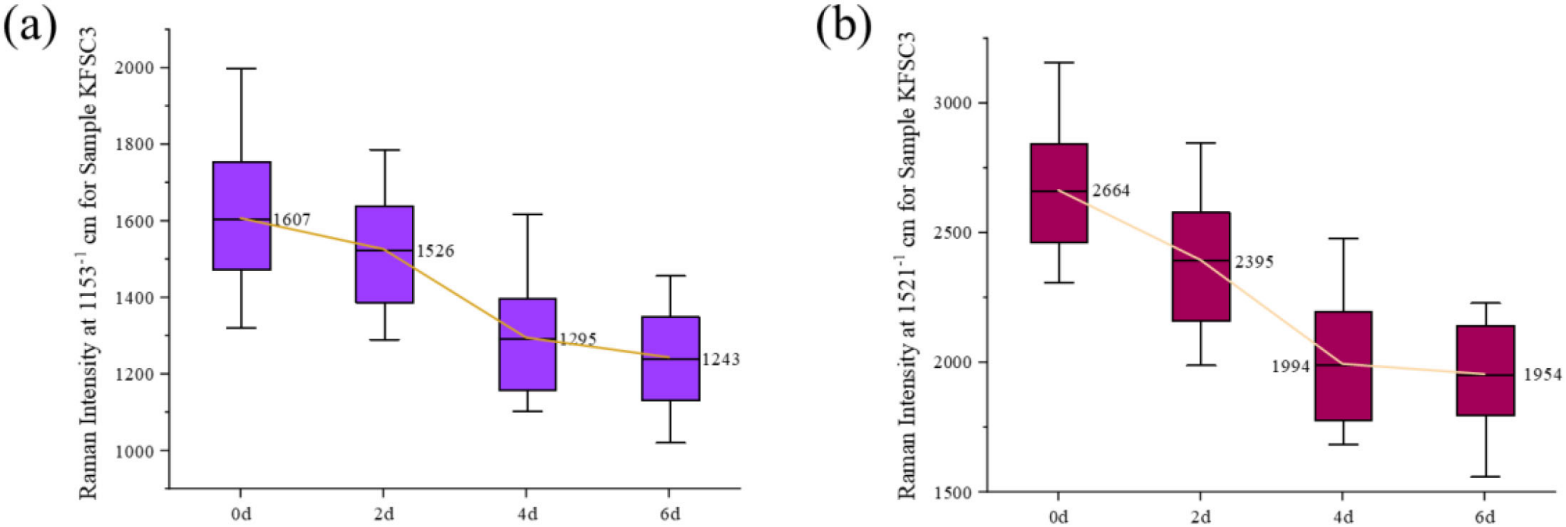
Intensity dynamics of key Raman peaks in resistant soybean leaves during SMV infection. **(a)** Peak at 1,153 cm^-^¹. **(b)** Peak at 1,521 cm^-^¹. Data are from the resistant cultivar KF at 0, 2, 4, and 6 days post-inoculation.

In general, the Raman spectra from soybean leaves of the 1138 and KF varieties exhibited broadly similar trends after inoculation with the SC3 virus. However, as can be seen from **Figure 1**, compared with the susceptible variety 1138, the Raman signal intensity of the resistant variety KF at 1,153 cm^-1^ and 1,521 cm^-1^ remained stable and did not appear to decay with the increase of infection degree. It demonstrates that during the process of virus infection in soybean leaves, defensive compounds of plants show significant dynamic regulation characteristics. **Figures 3** and **4** demonstrate the variation of Raman peak intensities at 1,153 and 1,521cm^-1^ for KF and 1138 cultivars under different infection times.

**Figure 3** shows the changes in the Raman spectra of leaf samples across all 1138 varieties at 0, 2, 4, and 6 days post-inoculation. The healthy leaf samples (0d) exhibited high-intensity Raman bands characteristic of carotenoids. However, the intensity of the carotenoid peak at 1,153 cm^-^¹ decreased significantly with the progression of infection, a trend that was particularly evident by the 6 d. This marked decrease suggests an acceleration of carotenoid degradation. Similarly, the Raman characteristic peak of carotenoids at 1,521 cm^-1^ also showed a synchronous decrease trend, indicating that the synthesis of carotenoids was inhibited or degraded with the progress of virus infection.

**Figure 4** presents the Raman spectral variation in leaf samples from all KF varieties at 0, 2, 4, and 6 days post-inoculation. The figure can obviously reflect the changes of leaf carotenoid content in healthy samples (0 d) and after 2 d, 4 d and 6 d of virus inoculation, which is the same as that of the 1138 variety. However, on day 6 after virus inoculation, the decrease in the intensity of the carotenoid Raman peak at 1,153 cm^-1^ and 1,521 cm^-1^ in leaves of the KF variety was slower than that of the 1138 variety. This may indicate that leaves of KF have a resistant response to virus infection, which can reduce the toxic effects of virus on plants by regulating metabolic pathways and enhancing the accumulation of defensive compounds (Sanaeifar et al., 2022).

### Determination of deep learning model construction

**Figure 5:**
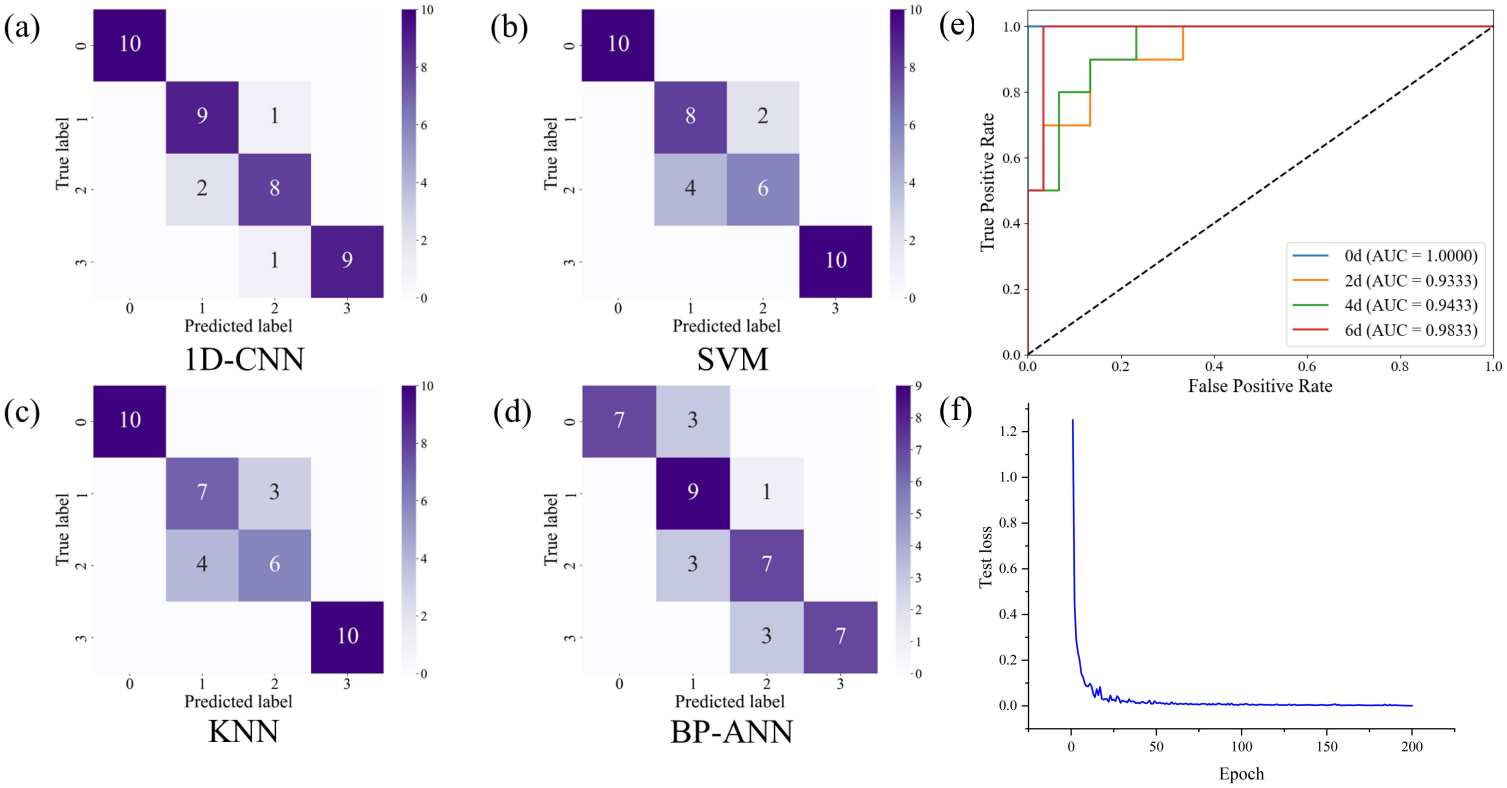
Confusion matrix plots of different models for 1138 soybean leaf prediction sets at healthy (0 d), 2 d, 4 d, and 6 d time points after virus infection. **(a-d)** Confusion matrices for the **(a)** 1D-CNN, **(b)** SVM, **(c)** KNN, and **(d)** BP-ANN models. **(e)** 1D-CNN model test set ROC curves at different infection stages (0 d, 2 d, 4 d, and 6 d) of Soybean Mosaic Virus. **(f)** Loss function change curve for the 1D-CNN model test set.

Before the machine learning and deep learning models was constructed, the preprocessed spectral data was divided into datasets. Specifically, the data were split into a training set and a test set in a 3:1 ratio. In this study, for the SVM model, a radial basis function (RBF) kernel was adopted to enable a nonlinear mapping of the original features into a higher-dimensional space. For the KNN model, after hyperparameters were optimized via grid search with cross-validation, the final class label of each test sample was determined by majority voting among its five nearest training samples in the high-dimensional spectral feature space, using Euclidean distance. For the BP-ANN model, the hidden layer comprised 20 neurons. The 1D-CNN model comprises two Conv1D layers with 16 and 32 filters, respectively, both using a kernel size of 3 and ReLU as the activation function. Each convolutional layer was followed by a MaxPooling1D layer with a pool size of 2 and a BatchNormalization layer to enhance feature extraction and improve training stability. The extracted features were then flattened and passed through a fully connected layer with 64 neurons and ReLU activation function, followed by a Dropout layer with a rate of 0.2 to reduce the risk of overfitting. Finally, a softmax activation function was employed in the output layer to classify the samples into four stages of disease progression. **Figure 5a-d** show the confusion matrix diagram of random samples of the test set established according to the above machine learning algorithm and the deep learning algorithm for four-classification training; **Figure 5e, f** show the ROC curve and loss function change curve of the 1D-CNN model test set.

After preprocessing with S-G smoothing and Air-PLS baseline correction, four classification models—1D-CNN, SVM, KNN, and BP-ANN—were developed to discriminate among healthy (0 d) and infected samples at 2, 4, and 6 days post-inoculation. The evaluation metrics of each model are presented in **Table 2**.

**Table 2:** Performance evaluation indicators of the four classification models on the testing dataset.

Models	Accuracy	Precision	Recall	F1-score
1D-CNN	0.9000	0.9045	0.9000	0.9011
SVM	0.8500	0.8542	0.8500	0.8485
KNN	0.8250	0.8258	0.8250	0.8246
BP-ANN	0.7500	0.8090	0.7500	0.7584

As shown in **Table 2**, evaluation of the four models’ performance indicators reveals that the 1D-CNN model outperforms the other three following data preprocessing. All indicators can reach above 0.9, achieving an ideal effect in the early disease detection of soybean leaves. As shown in **Figures 5e, f**, the AUC values of the 1D-CNN model in the test set for different stages of Soybean Mosaic Virus infection are all above 0.9, indicating high sensitivity and specificity at all time points. The loss function curve of the test set exhibits rapid convergence during training and stabilizes after approximately 50 iterations, suggesting that the 1D-CNN model can effectively classify soybean leaf samples at different infection stages.

SC3 virus infection can interfere with the normal metabolic activities of soybean leaf cells, disorganizing chloroplasts and reducing the content of photosynthetic pigments, and eventually inhibit the growth of soybean by reducing the efficiency of photosynthesis (Farber et al., 2019). SC3 virus inhibited carbohydrate metabolism in the process of infecting soybean leaves (Mandrile et al., 2019). This may account for the significant variation in Raman peak intensity. The developed deep learning model effectively captured these Raman peak intensity variations caused by SC3 infection. Consequently, the model demonstrated high proficiency in the early detection of SMV, achieving a superior recognition rate.

### PCA cluster analysis

In this study, PCA was performed on the Raman spectra of resistant and susceptible soybean leaves at different infection time points to observe the clustering trends of the two cultivars’ spectral profiles during various stages of infection.

**Figure 6:**
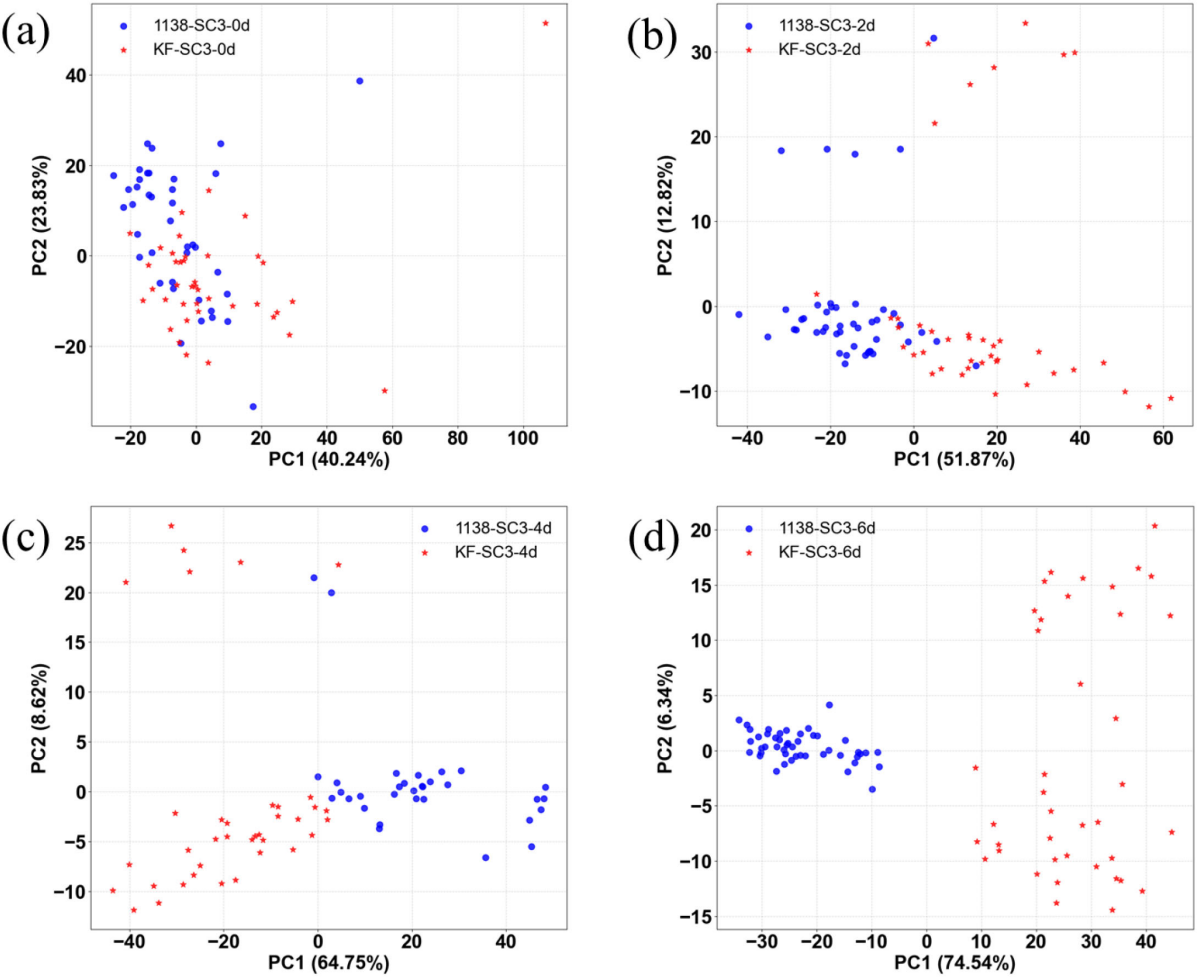
PCA plots of Raman spectra for resistant (KF-SC3) and susceptible (1138-SC3) soybean leaves at different infection time points. **(a)** 0 d (healthy). **(b)** 2 d **(c)** 4 d **(d)** 6 d.

**Figure 6** illustrates the principal component analysis (PCA) clustering patterns of Raman spectra for resistant (KF-SC3) and susceptible (1138-SC3) soybean cultivars at different infection time points (0 d, 2 d, 4 d, and 6 d). In **Figure 6a** (0 d, healthy stage), the samples from both cultivars showed extensive overlap in the PC1 – PC2 space, with a cumulative variance contribution of 64.07%, indicating highly similar initial physiological states and spectral profiles. As infection progressed, inter-cultivar differences gradually emerged and intensified. By 2 days post-inoculation (**Figure 6b**), the samples began to separate along the PC1 axis (variance contribution 51.87%), reflecting distinct early responses to infection. At 4 days post-infection (**Figure 6c**), clustering along PC1 became more distinct, with the explained variance rising to 64.75%, suggesting that mid-stage infection leads to further chemical differentiation between the cultivars. By the late infection stage (6 d, **Figure 6d**),the samples were clearly separated in the PCA space, with the variance contribution of PC1 further increased to 74.54% and cumulatively explaining over 80% of the spectral differences, indicating a fundamental divergence in the physiological states between susceptible and resistant plants. In summary, PCA clearly captured the transition from spectral homogeneity to heterogeneity between the two cultivars over the course of infection. Crucially, reliable differentiation could be achieved as early as 4 d. This demonstrates that Raman spectroscopy combined with PCA can sensitively detect early physiological changes under viral stress, providing a powerful analytical tool for non−destructive and rapid phenotyping of disease resistance in crops.

## Discussion

The results of this work demonstrate that combining portable Raman spectroscopy with data-driven modeling provides a robust and non-destructive strategy for the early identification of Soybean Mosaic Virus (SMV) infection. The most pronounced spectral variations were found in the carotenoid-related bands at 1,153 cm^-1^ and 1,521 cm^-1^, where resistant and susceptible cultivars displayed distinct temporal attenuation profiles. These spectral differences likely reflect alterations in host metabolic regulation and antioxidant activity induced by the viral infection. The slower decrease of carotenoid-associated signals in resistant cultivars indicates that resistance mechanisms mitigate pigment degradation and help preserve photosynthetic performance during infection, consistent with previously reported physiological observations in SMV-resistant soybean genotypes (Song et al., 2024).

Among the four classification algorithms evaluated, the 1D-CNN framework achieved the highest accuracy, highlighting its capability to capture complex nonlinear spectral-temporal dependencies that traditional machine learning models often fail to resolve. This finding underscores the significance of feature-learning approaches in plant pathology, where disease progression involves subtle biochemical changes dispersed across broad spectral ranges. Moreover, the clustering behavior revealed by PCA confirmed the dynamic divergence between resistant and susceptible cultivars over the infection timeline, reinforcing the interpretability and reliability of the Raman-based classification.

Compared with existing studies applying portable Raman spectroscopy and machine learning for early disease diagnosis in crops such as tomato and rice (Weng et al., 2021), this work provides new evidence supporting the feasibility of Raman-based phenotyping in soybean. The integration of portable spectroscopic sensing with deep neural architectures surpasses conventional discriminant models by offering higher sensitivity and temporal resolution under natural infection conditions. This methodological framework bridges biochemical detection and precision breeding by linking spectral fingerprints to phenotypic resistance characteristics.

This study develops a portable detection framework that integrates Raman spectroscopy with machine learning for early diagnosis of plant viruses. Unlike CRISPR/Cas-based detection, which requires nucleic acid extraction (Chen et al., 2019), the proposed method facilitates completely reagent-free and *in situ* analysis. In contrast to high-throughput sequencing, whose sensitivity is limited by viral nucleic acid abundance (Villamor et al., 2019), this approach enables pre-symptomatic diagnosis by probing early biochemical alterations in the soybean plants (e.g., Raman peak shifts specific to carotenoids) within four days post-inoculation. Whereas multispectral imaging identifies disease through macroscopic phenotypic changes (Singh et al., 2016), Raman spectroscopy yields complementary molecular-level fingerprint data. By integrating virus detection and resistance screening on a single field-portable platform, the present work advances intelligent in-field diagnostics and offers a practical framework for smart agriculture.

Future studies must account for additional variables that influence Raman spectral outcomes. Fluctuations in environmental conditions—including temperature, light intensity, and soil moisture—can alter key plant biochemical processes, notably the synthesis and breakdown of photosynthetic pigments. These changes manifest as shifts in spectral baselines and modifications to characteristic Raman peaks. Critically, such abiotic stress-induced spectral variations often closely resemble the signals produced by early-stage pathogen infection, thereby challenging the transferability of diagnostic models trained under controlled laboratory settings to real-world field conditions. Another major constraint lies in the inherently low signal intensity of portable Raman systems, which is further compromised in tissues with waxy surfaces or high moisture content, where target signals are frequently masked by fluorescence and noise. To address these limitations, the integration of eco-compatible, sensitive, and stable surface-enhanced Raman scattering (SERS) substrates with cross-cultivar transfer learning methodologies offers a promising pathway toward improved signal consistency and enhanced model robustness in diverse agricultural environments.

In summary, this study confirms the scientific feasibility of integrating Raman spectroscopy with deep learning for early detection of SMV infection and establishes a scalable analytical framework that unites optical phenotyping and artificial intelligence in crop protection. The proposed approach advances high-throughput, non-invasive, and intelligent monitoring strategies for plant viral diseases and provides a pathway toward data-driven resistance breeding and precision agriculture.

## Conclusion

In this work, portable Raman spectroscopy combined with advanced computational analysis was applied for the on-site and early identification of SMV infection. Raman spectroscopy enabled the detection of diagnostic spectral signatures characteristic of symptom onset at 4 days post-inoculation with the SC3 virus. The Raman bands associated with carotenoids at 1,153 cm^-1^ and 1,521 cm^-1^ gradually weakened as the infection advanced, whereas the resistant cultivars exhibited a slower reduction, indicating that resistance-related mechanisms mitigated the metabolic deterioration triggered by viral infection. Among the constructed classification frameworks, the 1D-CNN achieved the best recognition rate (90%), capturing the temporal evolution of spectral variations induced by infection. Moreover, PCA clearly revealed the progressive separation between resistant and susceptible samples in spectral space.

The portable Raman spectroscopy approach developed in this study, capitalizing on its non-destructive nature, minimal sample preparation, and field readiness, provides a practical tool for rapid, on-site diagnosis and early intervention in the fight against crop viral diseases. The early and accurate identification enabled by this technology allows for timely implementation of precision agricultural measures—such as roguing infected plants and optimizing crop rotation—prior to widespread disease epidemics. Collectively, this work presents a high-accuracy, non-invasive approach for early disease diagnosis and resistance screening. By mitigating yield losses and reducing reliance on preventive pesticides, it supports the transition towards more sustainable agricultural practices.

## Data Availability

The raw data supporting the conclusions of this article will be made available by the authors, without undue reservation.
